# Infrared Thermography as a Diagnostic Tool for the Assessment of Patients with Symptomatic Peripheral Arterial Disease Undergoing Infrafemoral Endovascular Revascularisations

**DOI:** 10.3390/diagnostics11091701

**Published:** 2021-09-17

**Authors:** Gladiol Zenunaj, Nicola Lamberti, Fabio Manfredini, Luca Traina, Pierfilippo Acciarri, Francesca Bisogno, Sabrina Scian, Raffaele Serra, Giulio Abatangelo, Vincenzo Gasbarro

**Affiliations:** 1 Department of Surgery, Unit of Vascular and Endovascular Surgery, University Hospital Arcispedale Sant’Anna of Ferrara, Via Aldo Moro 8, Cona, 44100 Ferrara, Italy; trainaluca@yahoo.it (L.T.); filippo.acciarri@gmail.com (P.A.); gsv@unife.it (V.G.); 2 Department of Neuroscience and Rehabilitation, Section of Sport Sciences, University of Ferrara, Via Ludovico Ariosto, 44121 Ferrara, Italy; nicola.lamberti@unife.it (N.L.); fabio.manfredini@unife.it (F.M.); frabis92@gmail.com (F.B.); sabrina.scian93@gmail.com (S.S.); giulio.abatangelo@edu.unife.it (G.A.); 3 Department of Medical and Surgical Sciences, Università Magna Graecia di, 88100 Catanzaro, Italy; rserra@unicz.it

**Keywords:** infrared thermography, peripheral arterial disease, critical limb ischaemia, ankle brachial index, percutaneous balloon angioplasty

## Abstract

Aim: The aim of this study was to evaluate the utility and reliability of temperature foot changes measured by infrared thermography (IRT) for the evaluation of patients with atherosclerotic peripheral arterial disease (PAD) before and after endovascular revascularisation. Methods: This is an observational prospective study carried out on symptomatic PAD patients. Evaluations consisted of a clinical examination, duplex scan with ankle–brachial index calculation (ABI) and IRT measurements with infrared camera FLIR-ONE connected to a smartphone with android technology. Locations on the foot sampled with IRT were the anterior tibial, pedal, posterior and arcuate arteries. Results obtained with IRT on the symptomatic foot were compared to the contralateral foot and with the ABI values obtained bilaterally before and 24 h after revascularisation. Results: Within one year, 40 patients were enrolled, among whom 87,5% suffered from critical limb ischaemia. In three patients, it was impossible to obtain ABI measurements because of ulcerations on the limb. Skin temperature changes obtained by IRT between the symptomatic limb and the contralateral limb had a mean difference of 1.7 °C (range: 1.1–2.2 °C), *p* < 0.001. There was a positive correlation between ABI and temperature values of the limb needed for treatment before revascularisation (*p* = 0.025; *r* = 0.36) and after revascularisation (*p* = 0.024, *r* = 0.31). The technical success rate was 100% in all cases, achieving a significant increase in temperature at all points of the foot analysed, with a median change of 2 °C (*p* < 0.001). Conclusion: IRT is a safe, reliable and simple application. It could be a valuable tool for the assessment of the clinical presentation and severity of foot blood perfusion in symptomatic PAD patients and the evaluation of the technical success of endovascular revascularisation. IRT might have a role in follow-up of revascularisation procedures.

## 1. Introduction

The incidence of peripheral artery disease (PAD) in patients with advanced stages is on the rise and is linked to the ageing of the population and the increasing incidence of cardiovascular diseases such as diabetes and chronic renal insufficiency [1]. The evolution of PAD towards critical stages exposes patients to a high risk for systemic complications correlated with limb ischaemia, and infections increase the mortality and limb loss rate [2].

The first evaluation of patients with PAD consists of clinical and non-invasive instrumental examinations, such as duplex ultrasound with measurement of the Ankle Brachial Index (ABI) [3], or more sophisticated measures for the ischaemic foot, such as transcutaneous oxygen pressure (TCPO2) [4], or near-infrared spectroscopy (NIRS) [5,6,7]. Infrared thermography (IRT) is an instrumental tool that is contact-free, non-invasive, low cost and simple to use. It measures the temperature dissipated from the foot skin, which depends directly on the quality of tissue blood perfusion. The reduction in tissue blood perfusion because of arterial vessel obstruction compromises cellular metabolism, thus decreasing heat production. Therefore, the foot skin temperature measured above these tissues might be dependent on the severity of atherosclerotic disease. IRT was previously applied in trials with PAD patients [8,9], or to determine the technical success of revascularization [10]. To the best of our knowledge, it has never been used to determine changes in the districts of the foot after endovascular treatment in the contest of critical limb threating ischaemia. The first objective of this study was to assess the efficacy and reliability of IRT in evaluating the clinical picture of symptomatic PAD patients compared to contralateral foot and ABI measurements obtained bilaterally. The second objective was to assess the efficacy and reliability of IRT in assessing the technical success of a revascularisation procedure.

## 2. Materials and Methods

This prospective observational study was carried out between March 2020 and March 2021. The Ethics Committee of Reference approved the study (ER.FE.2018.45.A). Written informed consent was obtained from all patients. To enhance clarity, this study is reported according to STROBE guidelines [11].

### 2.1. Subjects

Consecutive patients with symptomatic PAD were screened for possible inclusion in the study. The inclusion criteria were male or female PAD patients ages > 18 years old with atherosclerotic disease at stages 3, 4, 5 or 6 of the Rutherford classification and an indication for revascularisation with endovascular procedures in the arteries of the lower limb. Since, this is a preliminary study for the assessment of the role of IRT after endovascular limb revascularization, as the first choice of treatment at our institute, patient with indication for open procedures were excluded.

Patients were excluded if they presented with advanced critical limb ischaemia needing major amputation, no-option revascularization procedures, under vasodilating medications with prostaglandins, acute limb ischaemia or a PAD determined by a lower-limb artery aneurysm. Patients who needed open or hybrid procedures with both open and endovascular procedures were excluded. Patients who needed endovascular intervention at the aorta-iliac district were excluded as well. All patients at the admission underwent evaluation with collection of the vital parameters and those with body temperature more than 37 °C were excluded from the study. At baseline, demographic data, comorbidities and thermographic values of the patients were assessed and collected into an electronic dataset by an independent surgeon.

### 2.2. Thermographic Measurement Procedure

All patients underwent outcome measurements in their hospital rooms in a 21 °C temperature-controlled environment 12 to 24 h before peripheral endovascular revascularisation and 20 to 24 h after surgery. For all outcome measurements, patients rested in the supine position for at least 15 min, with the leg uncovered. Two practitioners, blinded to each other’s measurements, performed the following evaluations.

### 2.3. Foot Temperature

Foot temperature was assessed through a thermal camera (FLIR ONE-Pro, Flir System, Limbiate, Italy) connected to a smartphone equipped with the FlirOne Android© application.

**Figure 1:**
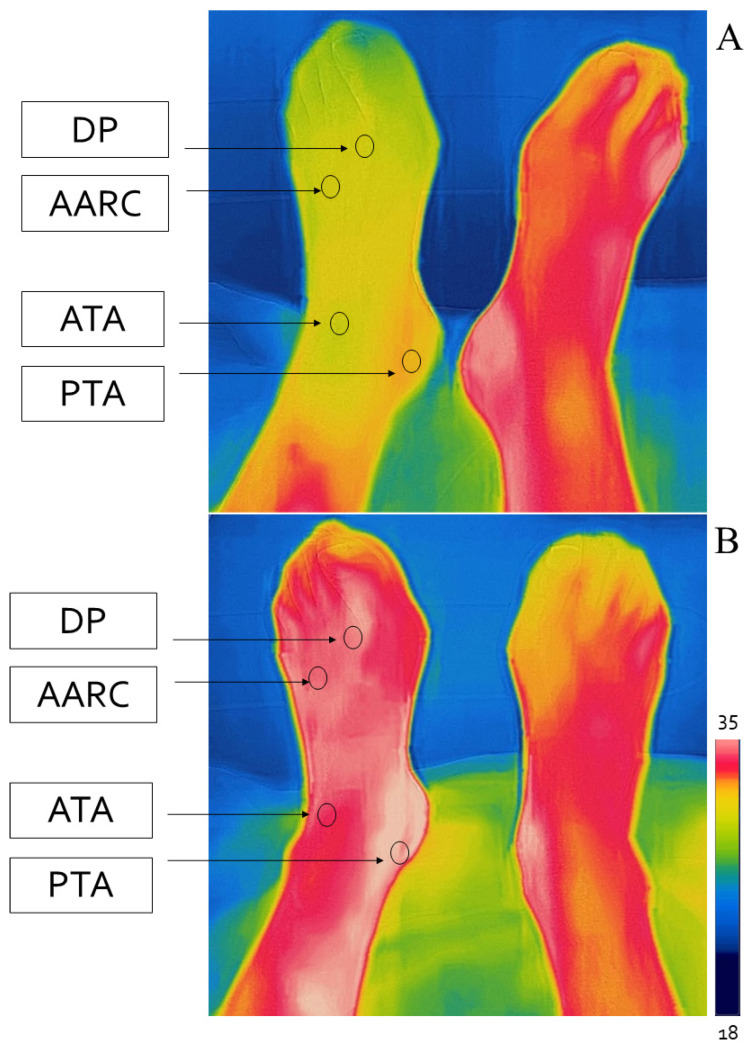
Sampling points of the foot temperature with infrared camera before (**A**) and after (**B**) revascularisation. DP: dorsalis pedis, AARC: arcuate artery, ATA: anterior tibial artery, PTA: posterior tibial artery. The scale ranges from 18 to 35 °C.

Temperature was assessed for both feet at four precise points corresponding to the posterior tibial artery under the medial malleolus, anterior tibial artery at the ankle, dorsalis pedis artery under the first metatarsal–phalangeal joint, and at the arcuate artery under the fourth metatarsal-phalangeal joint. A schematic representation of the sampling points is reported in Figure 1.

Data were automatically stored in a standard SD card and transferred to a laptop for data calculation and storage. For each point in each foot, data were collected twice, and the median values from those measurements were inserted in the proper dataset. Finally, a mean foot temperature value considering the four sampling points was also calculated.

### 2.4. Ankle–Brachial Index

The ABI was measured according to the published standard [11,12] with the patient lying in the supine position, using duplex ultrasound instrumentation (Philips S.p.A. Milano, Italy) and a standard blood pressure cuff. Blood pressure was measured and recorded at both the posterior and anterior tibial arteries of both limbs. Systolic and diastolic blood pressures were then assessed, and the highest value was used to calculate the AB index. The ankle–brachial index was defined as unreliable when values > 1.4 were obtained.

### 2.5. Statistical Analysis

The data distribution was verified by the Shapiro–Wilk test. Continuous variables are expressed as the mean ± standard deviation or median (interquartile range), and categorical variables are expressed as numbers and percentages. The differences in ABI and foot temperature were assessed through a paired-samples t-test, Wilcoxon test as appropriate. Correlations between the variables were obtained with Spearman’s rho. A *p* value ≤ 0.05 was considered statistically significant. Data were analysed using MedCalc Statistical Software version 19.8 (MedCalc Software., Ltd., Ostend, Belgium).

## 3. Results

Forty patients were enrolled in this study in a year’s time. The majority of patients (87.5%) presented with critical threatening limb ischaemia (Rutherford classification 4 to 6), and in three of them, it was not possible to determine the ABI value because of ulcerations on the leg and ankle. The demographics and clinical characteristics of the patients are reported in Table 1.

### 3.1. Baseline value of Foot Temperature

At baseline, significant differences between the two limbs (more impaired versus less impaired) were observed, with a mean difference of 1.7 °C (range: 1–2.2 °C). Data are reported in Table 2.

A progressively lower temperature was observed in the presence of a higher degree of PAD severity and in the more distal districts (dorsum of the foot) than in the ankle. (Table 3)

**Figure 2:**
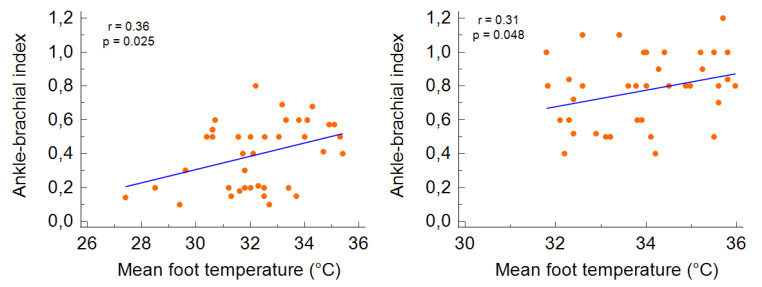
The correlation between the mean foot temperature and ABI before the revascularization in the more impaired foot (**left**) and the less impaired foot (**right**).

Before revascularisation, a positive significant correlation was observed for the mean temperature considering the four sampling points and the ABI value for the more impaired limb (*r* = 0.36; *p* = 0.025) and the contralateral limb (*r* = 0.31; *p* = 0.048) (Figure 2).

### 3.2. Revascularisation Procedures and Foot Temperature Changes

**Figure 3:**
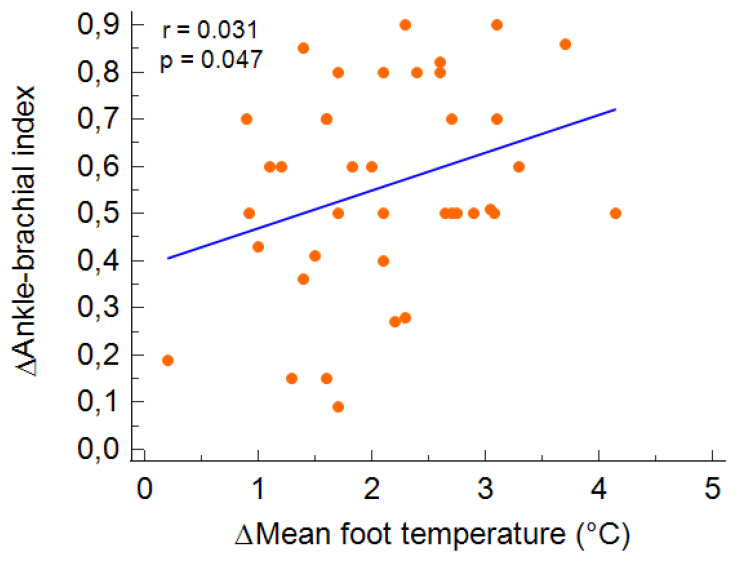
The correlation between the variations of mean temperature and ABI after the revascularization in the treated limb.

The revascularisation consisted of femoro-popliteal procedures with percutaneous balloon angioplasty (PTA) in 22 limbs with the need for a provisional stent in five cases, nine PTAs below the knee and nine cases of multiple procedures with PTA above and below the knee. In four patients, minor amputations of the toes were performed for dry gangrene, and in 6 patients, surgical debridement of the ulcerations was needed. The technical success rate was 100%, for recanalization of the targeted vessel without residual stenosis. In all patients, percutaneous closure devices were used and mobilised after 6 h, with no complications. No major cardiac or limb events were recorded. In all patients, a significant increase in the foot temperature was recorded in all districts sampled, with a mean increase of 2 °C (*p* < 0.001); the contralateral limb otherwise maintained stable temperatures (Table 4, Figure 3). After revascularisation, the ABI of the more impaired limb significantly increased (from 0.39 ± 0.19 to 0.93 ± 0.24; *p* < 0.001). Variation in the mean foot temperature was significantly positively correlated with the changes in ABI value for the more diseased limb (*r* = 0.31; *p* = 0.047) and was equally distributed over the four districts measured, without any significant difference. Figure 3.

There were no significant differences in terms of temperature changes with respect to the arterial segment treated, more precisely for above-the-knee procedures +1.9 °C, below the knee +2.3 °C, and multiple procedures at above and below the knee +1.7 °C.

Regarding the patients under medications with beta-blockers and calcium antagonists it was not possible to make any comparison because of the heterogeneity and the small number of the participants for each of the subgroups.

## 4. Discussion

Infrared thermography has been proposed as a diagnostic tool for the assessment of foot perfusion as a contact-free examination that is non-invasive and not influenced by the operator experience [13,14]. This exam could have a diagnostic role in evaluation of the diabetic foot, infectious processes and other inflammatory conditions, such as vasculitis, where an increase in the skin foot temperature is likely to be found [15,16]. Furthermore, IRT has been studied to assess the technical success of endovascular procedures for PAD patients [17].

In our study, we evaluated whether IRT could have a diagnostic role in assessing the clinical status of the foot before and after endovascular revascularisation in a setting where patients suffered mainly from critical limb ischaemia (Rutherford Categories 4–6). The data have been compared with ABI obtained bilaterally as a diagnostic tool validated and included in many guidelines for the management of patients suffering from PAD [17,18].

Our data confirmed a statistically significant relationship between the measurements obtained with IRT at each point of the foot and the clinical situation between the symptomatic foot and the contralateral foot (Table 2). In our study, where the majority of subjects were patients with critical limb ischaemia, the mean difference between the two limbs at the foot was 1.7 °C, approximately 6-fold superior to those reported in the literature for patients with intermittent claudication, namely, 0.3 °C [15]. These data confirm the relationship between the thermographic measurements and the severity of PAD. Our data show a progressive reduction in the temperature for the more distal foot areas, which becomes more evident for the higher Rutherford stages (Rutherford Categories 3 vs. 4, 5 and 6) because of a more severe compromission of the microvasculature of the forefoot.

Ilo et al. reported a comparison of thermographic data obtained from 164 PAD patients with those obtained from 93 healthy participants. The majority of their PAD sample was composed of claudicants, and the authors concluded that in these patients, the temperatures obtained were higher than those obtained from healthy control controls with normal ABI. According to them, this finding was attributed to vasodilation of small arterial vessels as a compensatory response to the overall reduction in blood flow [19]. This finding was not confirmed in our study and might be because of the small number of claudicants in our series. This might suggest a value of IRT for clinical assessment, especially in severe stages of atherosclerotic disease rather than for initial stages.

Considering that IRT for medical area application as a diagnostic tool is still at the beginning stages, threshold values to suggest a diagnosis of PAD are unavailable. For this reason, the temperature should be measured bilaterally to suggest a reduction in blood perfusion in the limb with lower temperature. However, considering other non-PAD causes that can determine an increase in temperature beside the data obtained by IRT, further evaluation of limb perfusion by a second specific diagnostic tool, such as ABI, should be mandatory [20].

Staffa et al. evaluated the efficacy of IRT for assessment of PAD patients before and after an endovascular procedure in a setting of claudicant patients [10]. Our data confirm the efficacy of IRT to assess the technical success of an endovascular procedure (in terms of increasing of foot temperature) and can be considered an external validation of their study. All points analysed showed significant increases in the temperature after (a successful) revascularisation, with a mean improvement of 2 °C, which is 4-fold higher than that reported for claudicants because of the high number of patients suffering from critical limb ischaemia in our study. On the contrary, in our series we did not have any case of technical failure regarding the target vessel intended to treat, therefore we do not have a group for comparison in this contest. However, the interpretation of both temperature and ABI changes are necessary to consider IRT as a tool to assess the technical success of an endovascular procedure. Furthermore, in our study we found that there were no significant differences in terms of temperature changes with respect to the arterial segment treated. Since, the main ischaemic target is the foot, this suggests that the main goal is to restore a successful direct inflow blood perfusion form the common femoral artery to the foot vessels and is independent of the anatomical segment treated.

In our opinion, IRT might offer some advantages over ABI in clinical assessment of the foot and the technical success of an endovascular procedure. There are some well-known factors that could influence the ABI measurement, such as the experience of the operator, calcifications of the vessel wall, occludent lesions, the depth of the arteries, the volume of the leg and foot and finally the presence of ulcerations at the area needed for measurements [21,22,23]. In the case of IRT, temperatures are obtained at several points of the foot regardless of the vessel used for calculation of ABI. Moreover, the depths of tibial arteries and ulcerations do not impede temperature measurements. For this reason, we believe that IRT might represent an adjunctive value for the non-invasive assessment of foot perfusion in PAD patients, overcoming the issues related to ABI.

As reported in the literature, thermography can detect abscess collection and a significant inflammatory reaction. In our study, the presence of tissue loss could be represented by points with higher temperatures, but we did not make this finding. The explanation could be linked to the overall decrease in foot temperature related to ischaemia considering the high number of patients suffering from the condition.

In our study, thermographic measurements were obtained by two independent operators without any difference in the data collected, confirming that IRT benefits from reliable reproducibility of the measurements performed by different operators [24]. Furthermore, thermal cameras for snapshooting are devices easily achievable at low cost, which are compatible with many smartphones currently on the market [25]. These features make IRT a pocket diagnostic tool, enabling caregivers to obtain important information about the clinical status of the foot that can be easily reproduced and shared while maintaining a high level of accuracy of the data.

This study has several limitations, such as the small number of participants, to validate the diagnostic role of IRT. Another limitation is the comparison of IRT data only with ABI results, and the comparison with data from different non-invasive examinations (as hyper spectral imaging or multifocal imaging) can contribute to better validating the diagnostic role of IRT. In general, peripheral oedema may represent a potential limitation for IRT measurements, but no patients under study presented this condition. Furthermore, many PAD patients may be under medication which induce a contraction of the small vessel as beta blockers or in the contrary medications which have a vasodilation effect such calcium antagonists. Moreover, in current smokers, nicotine induces a contraction of the small vessels musculature reducing the skin temperature. Considering the small sample all these drug-effects cannot be evaluated. Last, in our study, we included only patients undergoing endovascular revascularisations, but IRT could be used to assess the technical success of other revascularisation approaches, which might be another point to investigate.

## 5. Conclusions

IRT was revealed to be a reliable tool to assess the severity of atherosclerotic disease of the limbs of the same patient, demonstrating lower temperature values in the limb that was less perfused. The data obtained by IRT were sustained even from a positive correlation with ABI. IRT is safe, reliable and a simple application; it could be a worthy tool for the assessment of the clinical frame and severity of foot blood perfusion in symptomatic PAD patients. Furthermore, IRT seemed to be an efficient and non-invasive contact-free diagnostic tool to assess the technical success of revascularisation and might be useful in follow-ups.

## Figures and Tables

**Table 1:** Demographic data, comorbidities.

Demographics and Comorbidities	Patients (*n* = 40)
Age	76 ± 11
Male	27 (67.5%)
Female	13 (32.5%)
Current smokers	6 (15%)
Systemic hypertension	34 (85%)
Under beta blockers medication	21 (52.5%)
Under calcium antagonists medication	25 (62.5%)
Under beta-blockers and calcium antagonists	15 (37.5%)
Dyslipidaemia	19 (47.5%)
Diabetes mellitus	23 (57.5%)
Chronic renal failure	8 (20%)
CAD	19 (47.5%)
COPD	11 (27.5%)
Neoplasia	6 (15%)
Connetival disease	6 (15%)
Stroke	3 (7.5%)
Charlson index	3.79 ± 1.73
*ABI*	
ABI before revascularisation	0.31 ± 0.19
*Antomical segment*/Endovascular procedures	
*Femoro-popliteal*	22 (55%)
Balloon angioplasty	17 (42.5%)
Balloon angioplasty + SFA stenting	5 (12.5%)
*Below the knee* Balloon angioplasty	9 (22.5%)
*Above and below the knee* Balloon angiopasty Balloon angioplasty of BTKs + SFA stenting	9 (22.5%)
Minor amputations	4 (10%)
Wound debridements	6 (15%)
Stage of PAD	
Rutherford Category 3	5 (12.5%)
Rutherford Category 4	10 (25%)
Rutherford Category 5	17 (42.5%)
Rutherford Category 6	8 (20%)

ABI: values and clinical lower limb presentation according to the Rutherford classification, PAD: peripheral arterial disease, CAD: coronary arterial disease, COPD: chronic obstructive pulmonary disease, ABI: ankle–brachial index, SFA: superficial femoral artery, BTK: below the knee.

**Table 2:** Measurements obtained bilaterally with infrared thermography for both limbs.

	More Impaired Limb	Less Impaired Limb	*p* Value
Anterior tibial	32.7 ± 1.8	34.1 ± 1.4	<0.001
Posterior tibial	32.9 ± 1.6	34.1 ± 1.2	<0.001
Dorsalis pedis	31.7 ± 2.2	33.5 ± 1.6	<0.001
Arcuate	31.7 ± 2.1	33.6 ± 1.5	<0.001
Mean value	32.2 ± 1.8	33.9 ± 1.3	<0.001

The mean value is calculated as the mean of the four sampling points. Values are expressed as °C.

**Table 3:** The relationship between the clinical stage according to the Rutherford classification and the temperatures obtained from different foot locations.

	Grade 3 (*n* = 5)	Grade 4 (*n* = 10)	Grade 5 (*n* = 17)	Grade 6 (*n* = 8)	*p* Value
Anterior tibial	33.9 ± 1.8	32.6 ± 1.4	32.9 ± 1.9	31.7 ± 1.3	0.15
Posterior tibial	33.6 ± 1.9	32.5 ± 1.4	33.3 ± 1.4	31.9 ± 1.3	0.08
Dorsalis pedis	33.9 ± 2.1	31.8 ± 2.0	31.4 ± 1.6	30.2 ± 2.1	0.016
Arcuate	34.0 ± 1.7	31.8 ± 2.0	31.5 ± 1.6	30.0 ± 2.0	0.007
Mean value	33.9 ± 1.9	32.2 ± 1.7	32.4 ± 1.7	30.9 ± 1.4	0.016

Values are expressed as °C.

**Table 4:** Global and specific district temperature changes before and after limb revascularisation.

	More Impaired Limb			Less Impaired Limb		
	Before	After	*p* Value	Before	After	*p* Value
Anterior tibial	32.7 ± 1.8	34.6 ± 1.6	<0.001	34.1 ± 1.4	34.6 ± 1.6	0.35
Posterior tibial	32.9 ± 1.6	34.8 ± 1.5	<0.001	34.1 ± 1.2	34.6 ± 1.7	0.41
Dorsalis pedis	31.7 ± 2.2	34.0 ± 1.9	<0.001	33.5 ± 1.6	34.1 ± 1.6	0.22
Arcuate	31.7 ± 2.1	34.0 ± 2.0	<0.001	33.6 ± 1.5	34.1 ± 1.6	0.44
Mean value	32.2 ± 1.8	34.3 ± 1.7	<0.001	33.9 ± 1.3	34.4 ± 1.5	0.52

Values are expressed as °C.

## Data Availability

Data are available upon request to the corresponding author.
